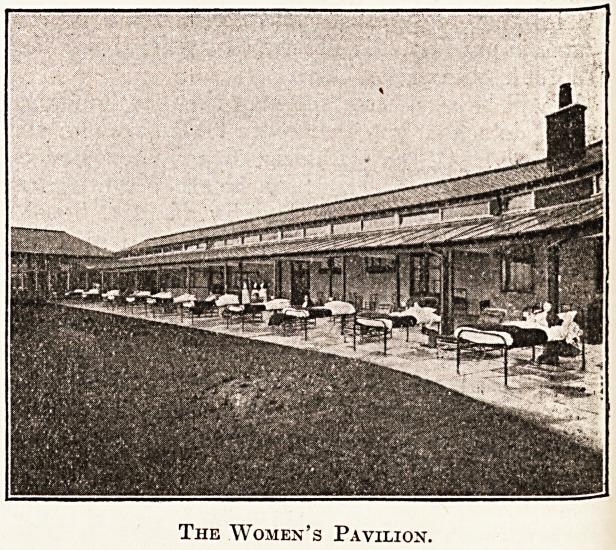# City Sanatorium, Birmingham

**Published:** 1915-05-29

**Authors:** 


### HOSPITAL ARCHITECTURE AND CONSTRUCTION.

### City Sanatorium, Birmingham.

We publish to-day a block plan illustrating the whole of the buildings which comprise the sanatorium for tuberculosis established by the Corporation of Birmingham, together with details showing the internal arrangement and construction of the women's pavilion, the children's pavilion, and the children's observation block.

It will be seen from the plan that the buildings are all detached, and that seven blocks are old, six being new. Of the old blocks, five are occupied as wards for men. The new blocks comprise an administrative block, a laundry, boiler house, women's pavilion, children's pavilion, and children's observation block.

The administrative block provides accommodation for the medical staff, nursing staff, and servants; and the kitchen offices for the whole institution.

The laundry building is planned on the usual lines of a big institution, and contains also a disinfecting house fitted with a steam disinfector opening into two separate rooms.

The boiler house is fitted with one steam boiler, space being left for the addition of a second boiler in the future.

The women's pavilion provides accommodation for seventy-two beds in two groups of thirty-six divided by the dining hall. These groups of thirty-six are divided again into two by the duty room, and these again into groups of eight by a glazed screen.

The beds are placed in rooms which are entirely open on the south side, and in front is a wide verandah which makes the total available depth from front to back about 22 feet. The roof over the inner part is carried up high enough to allow cross ventilation above the roofs at front and back. The patients in this block will be kept out on the terrace night and day when the weather is sufficiently settled. During rainy or unsettled weather the beds will be under the glass roof, and in stormy weather will be wheeled back to the wall out of reach of rain or snow.

The Block Plan of the Sanatorium.

At the back are dressing rooms in the proportion of one to four patients, a bathroom to each group of eight patients, four w.c.s and one for nurses; attached to each duty room is a sink room and pantry, and each main group of thirty-six is provided with a linen store, a housemaid's sink and orderly room, and a drying room for clothes.

City Sanatorium, Yardley Road, Birmingham.

On one side of the dining hall is a serving room, on the other a china pantry.

No provision is made for warming any part of the building except the dining hall, which has two fireplaces; it is intended to utilise this room also for concerts and services.

The children's block is planned very much on the same lines as the women's pavilion; the space for beds is much deeper and contains two rows of beds. The dressing rooms and bathrooms are arranged so that several children can be bathed and dressed at the same time; in other respects the auxiliary offices are similar to those in the women's pavilion.

The children's observation block contains ten cubicles separated by glazed partitions, partly screened in on the front side, and having no open terrace in the front of the glass verandah.

The Sanatorium Dining-hall.

The children are kept here for a fortnight before being sent to the main block; by this arrangement diagnosis is made certain and the possibility of importing other infections greatly diminished.

The offices attached to this block comprise a duty room, sterilising and wash-up room, pantry, nurses' lavatory and w.c., sink room, linen store, bathroom and patients' w.c. There is also a portable bath which stands in a recess in the corridor.

The special feature of this sanatorium is the absolute open-air construction of the wards, there being no means whatever of closing the front. The adoption of this method is due partly to experience at Birmingham and partly to the example of Hunt's Sanatorium at Barchurch, where children have been treated in this way with great success for many years.

The Women's Pavilion.

The architects of the sanatorium are Messrs. Ward, of Birmingham.

## Figures and Tables

**Figure f1:**
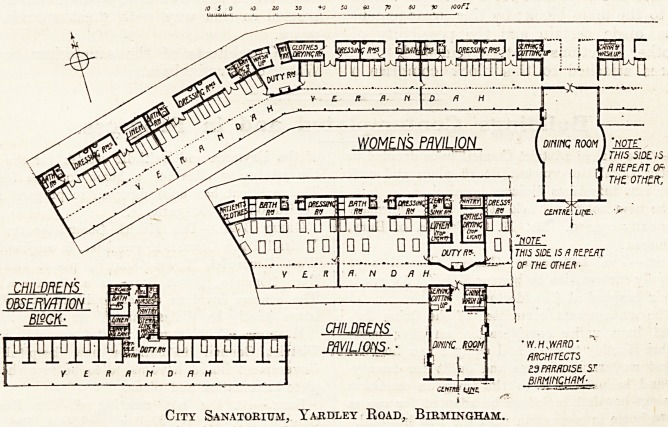


**Figure f2:**
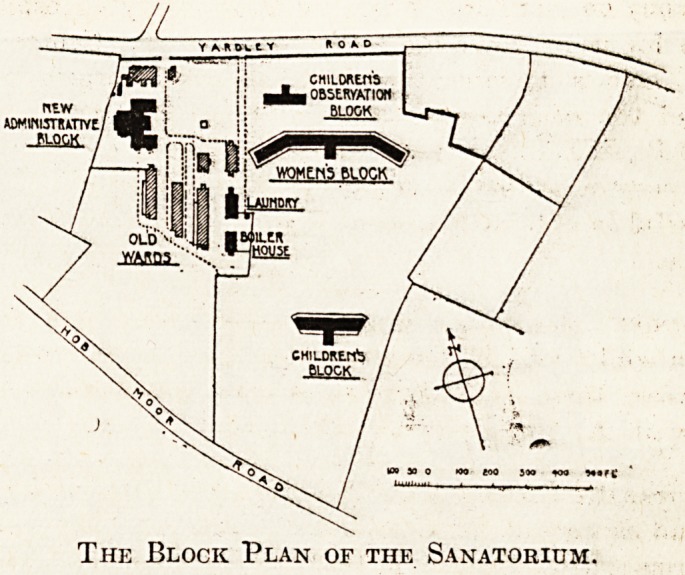


**Figure f3:**
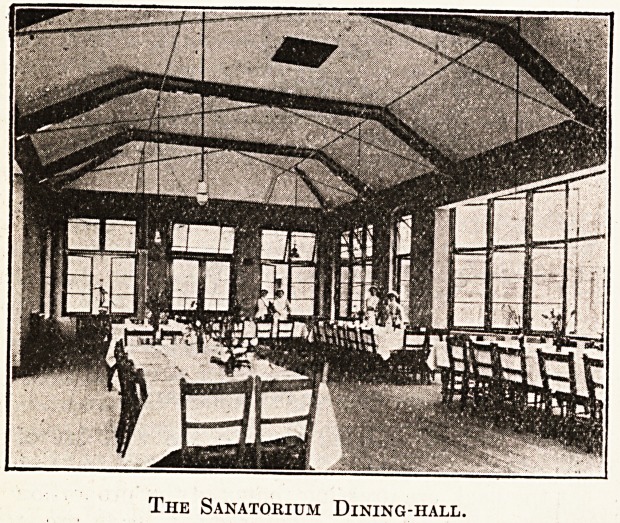


**Figure f4:**